# State-of-the-Art Definitive Femoropopliteal Lesion Treatment: A Case-Based Systematic Approach

**DOI:** 10.3390/jcdd13040150

**Published:** 2026-03-28

**Authors:** Grigorios Korosoglou, Nasser Malyar, Andrej Schmidt, Michael Lichtenberg, Gerd Grözinger, Dittmar Böckler, Christian A. Behrendt, Erwin Blessing, Ralf Langhoff, Thomas Zeller, Christos Rammos

**Affiliations:** 1 GRN Hospital Weinheim, Cardiology and Vascular Medicine, 69469 Weinheim, Germany; 2 GRN Hospital Eberbach, Cardiology and Vascular Medicine, 69469 Weinheim, Germany; 3 Department of Cardiology I-Coronary and Peripheral Vascular Disease, Heart Failure, University Hospital Muenster, 48149 Muenster, Germany; nasser.malyar@ukmuenster.de; 4 Division of Angiology, University Hospital Leipzig, 04103 Leipzig, Germany; dr.andrej.schmidt@gmail.com; 5 Vascular Center, Klinikum Arnsberg, 59821 Arnsberg, Germany; klichte@gmx.net; 6 Department of Radiology, SLK-Kliniken Heilbronn GmbH, 74078 Heilbronn, Germany; gerd.groezinger@slk-kliniken.de; 7 Department of Vascular and Endovascular Surgery, University Hospital Heidelberg, 69120 Heidelberg, Germany; dittmar.boeckler@med.uni-heidelberg.de; 8 Department of Vascular and Endovascular Surgery, Asklepios Clinic Wandsbek, Asklepios Medical School, 22043 Hamburg, Germany; behrendt@hamburg.de; 9 University Heart and Vascular Center, Department of Angiology, University Hospital Hamburg-Eppendorf, 20246 Hamburg, Germany; e.blessing@uke.de; 10 Department of Angiology, Center for Internal Medicine I, Campus Clinic Brandenburg, Brandenburg Medical School Theodor Fontane, 14770 Brandenburg an der Havel, Germany; ralf.langhoff@sankt-gertrauden.de; 11 Department of Angiology and Vascular Medicine, Sankt Gertrauden Hospital, Humboldt University Berlin, 10713 Berlin, Germany; 12 Department of Interventional Angiology, University Hospital Freiburg/Bad Krozingen, 79189 Bad Krozingen, Germany; thomas.zeller@uniklinik-freiburg.de; 13 Department of Cardiology and Vascular Medicine, West German Heart and Vascular Center, University of Duisburg-Essen, 45141 Essen, Germany; christos.rammos@uk-essen.de

**Keywords:** definitive lesion treatment, case-based approach, femoropopliteal lesions, chronic total occlusions (CTOs), drug-coated balloon angioplasty (DCB), bare-metal stents (BMSs), drug eluting stents (DESs), interwoven stents

## Abstract

After vessel preparation, using different strategies such as balloon angioplasty, specialty balloons, atherectomy or intravascular lithotripsy, definitive treatment has emerged as a key feature in endovascular treatment strategies. Based on current guidelines, endovascular treatment is the most common treatment option in patients with claudication. In patients with chronic limb-threatening ischemia (CLTI), on the other hand, the best treatment modality, including bypass surgery and endovascular revascularization, needs to be selected by an interdisciplinary team, focusing on individual anatomic and patient-specific characteristics, on the availability of a vein graft and on cardiovascular and other comorbidities of the patients. With endovascular therapy, currently, a plethora of options are available for the treatment of femoropopliteal lesions, which are increasingly gaining in complexity. Therefore, a practical systematic case-based approach, entailing contemporary treatment options, like drug-coated balloon (DCB) angioplasty tools, self-expanding bare-metal stents (BMSs), drug-eluting stents (DESs), interwoven stents and covered stents, is crucial. Generally, most endovascular operators adhere to the ‘leave nothing behind’ concept, meaning that, after proper lesion preparation, lesions can be treated with DCBs, avoiding the implantation of permanent metallic implants. However, in the case of severe dissections or significant recoil, stent implantation becomes necessary to achieve adequate limb perfusion. The selection between long versus spot stenting and the different stent options depends on the current scientific evidence, guidelines and expert opinion statements. An interdisciplinary expert consensus was recently compiled on how these modalities should be used in specific lesions and patients in the femoropopliteal segment. Herein we present a practical case-based approach, which is based on this algorithm and aims at harmonization of endovascular treatment strategies in daily practice and ultimately at further improvements in limb and patient outcomes.

## 1. Introduction

Endovascular treatment of peripheral artery disease (PAD) has continuously evolved over the past decades and is currently the most common first-line option for treatment of acute and chronic arterial lesions, being widely used in various medical and surgical disciplines [1,2,3]. After preprocedural imaging by duplex ultrasonography or computed tomography angiography and planning of the most appropriate access site, lesion crossing becomes necessary, which in chronic total occlusive (CTO) lesions may require the use of antegrade wire escalation or/and advanced retrograde and bidirectional crossing techniques [4,5]. Based on lesion length, calcification and subintimal versus intraluminal guidewire passage, lesion preparation strategies become available, which aim to remove plaque, especially calcific tissue or an organized thrombus from the vessel wall, thereby improving vessel compliance and enhancing subsequent drug delivery to the vessel wall during definitive lesion treatment [6]. However, in the case of severe dissections or significant recoil, the implantation of permanent metallic implants becomes necessary. Although the implantation of drug-eluting stents is mentioned with current national guidelines [7], there is currently no generally accepted consensus on the choice and use of definitive treatment strategies based on patient- and lesion-specific characteristics. However, experts from different disciplines recently demonstrated a high grade of agreement for the application of lesion preparation and definitive treatment strategies for the endovascular revascularization of femoropopliteal lesions and CTOs [3]. Herein, a case-based approach is presented, with different lesion treatment options in selected patient- and lesion-specific scenarios.

## 2. Drug-Coated Balloon Angioplasty

Findings from randomized controlled trials (RCTs) have shown that the use of paclitaxel-coated balloons exhibits clear advantages over conventional balloon angioplasty in terms of reintervention and primary patency rates compared to uncoated balloon angioplasty [8,9,10]. Based on the overwhelming body of evidence derived from RCTs and subsequent meta-analyses, the adjunctive use of paclitaxel emerged as the standard-of-care primary treatment for femoropopliteal lesions [8]. However, a benefit regarding hard clinical endpoints such as improved amputation-free survival (AFS) has not been demonstrated for DCB so far. In addition, in 2018, a pooled meta-analysis of RCTs showed an increase in 5-year all-cause mortality in patients treated with paclitaxel-coated devices [11]. However, with the subsequent availability of patient-level data from the studies included in the original meta-analysis, the magnitude of the mortality signal steadily decreased, while real-world data from registries could not confirm this signal even over a longer follow-up period [12]. In addition, the updated report from the SAFE-PAD study (168,553 patients, 70,584 (41.9%) treated with drug-coated devices) demonstrated that, up to 6.3 years, drug-coated devices are not associated with increased mortality [13]. Based on these findings, DCBs are once again part of the standard treatment for femoropopliteal lesions, with a high grade of agreement for their use among experts from various disciplines [3]. However, the Swedish Drug-Elution Trial in Peripheral Arterial Disease 2 (SWEDEPAD 2) trial [14] recently reported that quality of life after 1 year did not differ between patients with claudication treated with paclitaxel-coated versus uncoated devices. All-cause mortality, on the other hand, was higher in patients randomly assigned to paclitaxel-coated devices after 5 years, thus again raising a warning signal against the routine use of paclitaxel in patients undergoing infrainguinal endovascular revascularization. In addition, the SWEDEPAD 1 trial assessed the safety and efficacy of paclitaxel-coated devices in patients with CLTI [15]. In this trial, no significant differences were noted in the rate of ipsilateral major amputations and mortality rates, whereas reintervention rates were lower in patients treated with paclitaxel-coated devices after 1 year. Notably, treated lesions were in the femoropopliteal vascular segment only in 52.7% of cases, whereas in the remaining patients, lesions were in both femoropopliteal and infrapopliteal vessels or only in infrapopliteal arteries, where paclitaxel-coated balloons generally do not exhibit higher effectiveness compared to uncoated devices. In addition, previous studies noted the slow flow phenomenon in patients treated with DCB angioplasty. This finding may be associated with paclitaxel flaking and particle embolization during the angioplasty procedure and may be more especially relevant in patients with CLTI, CTO lesions, and lesions treated with several/long DCBs and in patients with pre-existing poor tibioperoneal run-off [16]. In addition, this phenomenon may be associated with poorer limb outcomes, including higher reintervention and amputation rates.

The above-mentioned safety concerns with paclitaxel-coated balloons, as well as the wide experience with sirolimus in the coronary space, contributed to the development of sirolimus-coated balloons dedicated to the treatment of PAD. Currently, two sirolimus-coated balloons have been approved for PAD treatment, including the Selution SLR™ and the Magic-Touch™ [17]. The Selution SLR™ combines the use of an amphipathic lipid cell adherence coating with biodegradable sirolimus micro-reservoirs to increase drug uptake into the arterial wall. Encouraging 6-month safety and effectiveness results have been reported in a recent single-arm trial, where Selution SLR™ was used for the treatment of patients with femoropopliteal lesions [18]. The MagicTouch™, on the other hand, employs the use of phospholipid to achieve 100% sirolimus sub-micron particle coating on its balloon surfaces, allowing for controlled drug delivery into the arterial wall [19]. The efficacy of sirolimus-coated balloons was initially limited by their slower spread within the arterial wall, reducing retention levels and resulting in rapid dilution and subtherapeutic treatment [17,19]. These limitations seem to be overcome, however, based on technologic advancements.

**Figure 1:**
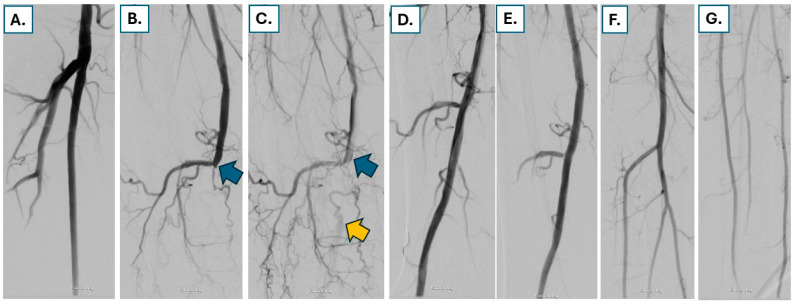
A 72-year-old male patient presented with severe claudication RC 3 due to a relatively short occlusion of his distal right superficial femoral artery (SFA) ((**A**), blue arrow in (**B**,**C**)) with flow reconstitution in the popliteal artery (orange arrow in (**C**)). After antegrade guidewire passage and uncoated balloon angioplasty, which was followed by sirolimus-coated balloon angioplasty, a good angiographic result without severe dissections (**D**,**E**) and with good flow to the foot can be observed (**F**,**G**).

Current ongoing studies focus on the safety and effectiveness of PCB and SCB for the treatment of femoropopliteal disease. In this regard, preliminary results of the SIRONA RCT were reported [20]. In the study, 482 patients with Rutherford category (RC) 2–4 femoropopliteal artery disease were enrolled in 25 clinical sites in Germany and Austria. Patients were randomized at a 1:1 ratio to receive angioplasty with either a sirolimus-coated or a paclitaxel-coated balloon. Preliminary results of the study showed that the sirolimus-coated MagicTouch (Concept Medical) balloon is non-inferior compared to paclitaxel-coated balloons regarding primary safety and efficacy endpoints. An example of a patient with RC 3 claudication treated with sirolimus-coated balloon angioplasty after uncoated angioplasty with a good angiographic result is shown in Figure 1.

## 3. Self-Expanding Bare-Metal Stents (BMSs) and Interwoven Stents

The use of self-expanding BMSs is associated with better acute angiographic results and improved patency compared to uncoated balloon angioplasty. However, the development of in-stent restenosis, which can lead to complete stent occlusion, is not uncommon. Especially in longer and calcified femoropopliteal lesions, the long-term patency of BMSs is relatively low, especially over long follow-up periods [21]. The primary patency rates were 73%, 64%, 47%, and 33% respectively at 1, 3, 5, and 7 years after BMSs in the femoropopliteal segment. After 7 years, freedom from reintervention was 47%, and the amputation-free survival was 73%. Increased amputation rates were noted in patients with diabetes mellitus, whereas involvement of the popliteal artery predicted increased rates of reinterventions. Furthermore, studies have found a higher patency rate with spot BMS implantation compared to long stenting [22]. Hereby, long stenting was an independent predictor of restenosis. Compared to spot stenting after adjustment of other clinical and anatomical variables, long stenting, especially when stent placement extended to the P2 or P3 segment of the popliteal artery, was independently associated with a 7.5-fold risk for restenosis.

**Figure 2:**
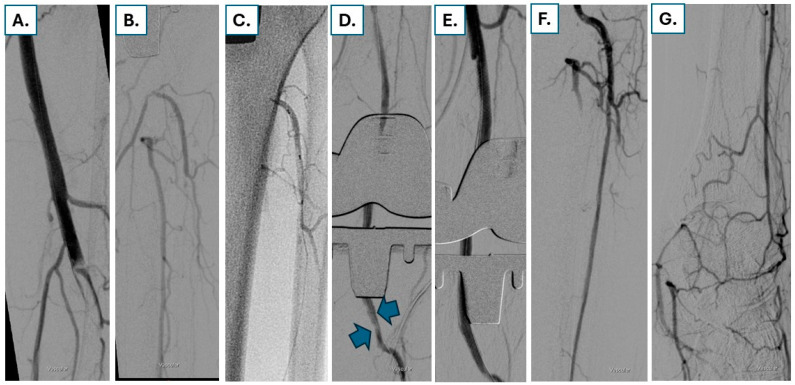
A 78-year-old male patient presenting with resting pain RC 4 due to chronic occlusion of the distal SFA and the popliteal artery (**A**) and with flow reconstitution in the infrapopliteal vessels (**B**). The antegrade guidewire crossing failed since the wire remained subintimal. In addition, angiographic view restrictions are present after artificial knee joint replacement. After failed antegrade crossing, distal puncture is performed at the proximal anterior tibial artery (**C**), enabling retrograde wire passage. After guidewire externalization, balloon angioplasty is performed, leading to antegrade flow but also to severe dissections and recoil (blue arrows in (**D**)). After non-compliant balloon angioplasty, two Supera^TM^ stents are implanted (**E**), restoring direct brisk blood flow to the dorsal pedal artery (**F**,**G**).

A special form of self-expanding BMS is the so-called interwoven stent (Supera^TM^ stent, Abbott Cardiovascular). Due to its specific architecture, the radial force and flexibility of this stent are markedly increased compared to conventional nitinol stents. This allows the interwoven stent to better adapt to biomechanical forces and exhibit greater resistance to recoil and stent strut fractures. This is particularly important in mobile femoropopliteal segments, such as the popliteal artery. Despite the fact that the Supera^TM^ vascular mimetic implant (Abbott Cardiovascular) has been in clinical use for many years, studies are limited, particularly regarding long-term results, and rather refer to short follow-up periods, while no comparative studies with other devices have been available so far [23]. Furthermore, when implanting the Supera stent in heavily calcified lesions, prior preparation of the lesion with non-compliant balloons is essential to avoid stent under-expansion due to elongation, which cannot be reversed by post-dilatation and can lead to stent failure due to in-stent restenosis or thrombosis. The angiographic images of a patient presenting with RC 4 due to chronic occlusion of the popliteal artery are shown in Figure 2. After subintimal guidewire passage from retrograde and uncoated angioplasty, severe dissections and re-coil were noted in the popliteal artery. After non-compliant balloon angioplasty, two Supera^TM^ stents were implanted, restoring direct blood flow to the foot of the patient.

## 4. Drug-Eluting Stents (DESs)

DESs combine the advantages of BMSs with anti-restenotic properties. A polymer enables extended paclitaxel elution into the vessel wall. Two paclitaxel-eluting stents are currently available on the market: the polymer-free Zilver PTX platform (Cook) and the polymer-coated Eluvia DES platform (Boston Scientific).

In a large-scale RCT (EMINENT study), the Eluvia DES demonstrated a significantly higher patency rate after one year compared to various BMSs [24]. However, the EMINENT study involved a selected cohort with a low percentage of long and severely calcified lesions, so the results are not necessarily comparable to real-world PAD cohorts. Furthermore, the interwoven Supera^TM^ stent was used in the BMS arm in only 17.3% of cases. In another RCT (IMPERIAL study), the Eluvia DES demonstrated greater freedom from reintervention after two years compared to the Zilver PTX stent [25]. Due to the encouraging data for DES, its use is highlighted in the current national PAD guidelines [7]. However, there is currently no reimbursement for the use of DESs in Germany. In another randomized study with 150 patients, which compared the Zilver PTX DES with DCB, a trend towards a better patency rate of the DES was shown over an observation period of 36 months, but the result did not reach statistical significance (54% versus 38%, *p* = 0.17) [26]. Interestingly, experimental data previously compared the long-term vascular healing responses of healthy swine iliofemoral arteries treated with a polymer-free paclitaxel-eluting stent (Zilver PTX) versus a fluoropolymer-based paclitaxel-eluting stent (Eluvia) [27]. This study demonstrated that prolonged paclitaxel release in the presence of a permanent polymer (Eluvia) may contribute to the differential vascular responses, including medial layer disruption and aneurysmal vessel degeneration. Although the clinical significance of these findings is still not completely clear, these data might guide operators in differentiating their use in daily practice. Recently, the REALDES trial showed comparable primary patency and freedom from clinically driven TLR after three years in patients with femoropopliteal disease. However, in patients with restenosis, the incidence of in-stent occlusion was statistically significantly higher with Eluvia (57.7%) than with Zilver PTX (29.2%) (*p* = 0.04) [28]. In addition, the prospective, multicenter, randomized SPORTS study (Sequent Please Drug Coated Balloons Versus Primary Stent Application in Long SFA Lesions; NCT03332264) is underway, comparing DCB vs. bare-metal or the Eluvia DES in patients with femoropopliteal lesions ≥13 cm in length. Preliminary results from a conference presentation show that the Eluvia DES is superior to a combination of DCB and BMS use in the femoropopliteal segment [29]. Based on preliminary results, SPORTS, DES provided superior outcomes versus a BMS and DCB, measured by freedom from TLR up to 12 months. The DCB arm reported a 58% bailout stent rate and showed non-inferior results compared to BMS. The results of this study could provide further arguments for the wider use of DESs in femoropopliteal lesions. In the recently published Delphi consensus, DESs reached a higher grade of agreement after a DCB for the definitive treatment of femoropopliteal lesions [3].

## 5. Covered Stents

For long and calcified femoropopliteal lesions, the use of self-expanding, heparin-bound, covered stents represents another alternative treatment option. These devices have shown encouraging long-term results in single-arm studies and improved patency rates compared to BMSs in randomized controlled trials. In the intention-to-treat group, the 24-month primary patency rates were 63.1% for covered stents versus 41.2% for BMS (*p* = 0.04), whereas freedom from TLR was 79.4% versus 73.0% (*p* = NS) [30]. In addition, covered stents may represent a viable treatment strategy in patients with diffuse in-stent restenosis (ISR) and stent occlusions (Tosaka II and III lesions). In this respect, the RELINE RCT demonstrated the superiority of covered stent placement versus balloon angioplasty in the femoropopliteal segment [31]. In the recently published Delphi consensus, agreement for the use of covered stents was, however, relatively low for the treatment of both de novo and ISR lesions among expert operators [3]. Notably, the best grade of agreement accompanied, however, by an only weak recommendation could be reached for the treatment of Tosaka II and III lesions.

**Figure 3:**
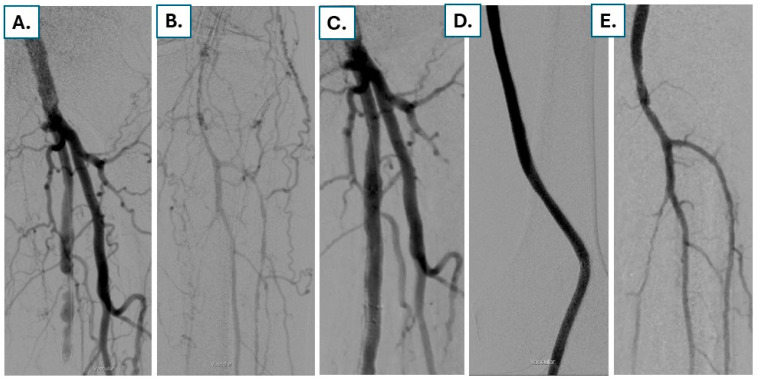
An 84-year-old male patient with gangrene RC6 due to long occlusion of the SFA and the popliteal artery (**A**) and faint contrast filling in the infrapopliteal arteries (**B**) after failed stenting and failed open surgery in the past. After crossing of the long occlusion and debulking using rotational thrombectomy, covered stents are implanted, resulting in a good angiographic result (**C**,**D**) with brisk flow to the infrapopliteal arteries (**E**).

An example of a patient with RC 5 due to long stent occlusion of the SFA and popliteal artery treated with covered stents with a good angiographic result is shown in Figure 3.

## 6. Algorithm for Definitive Lesion Treatment and Patient-Specific Considerations

Overall, although the current body of evidence suggests that DCBs improve outcomes versus POBA, their effectiveness as standalone tools in complex femoropopliteal disease remains unclear, where the optimal treatment option remains undefined and needs to be considered based on patients and lesion-specific parameters. DESs might improve the outcomes of endovascular therapy compared to primary scaffolding with BMSs; however, no large-scale studies so far have compared DESs with interwoven/biomimetic devices. In the recent BEST-SFA trial, including 120 patients with symptomatic (RC 2–4) PAD due to complex femoropopliteal lesions, patients were randomly assigned to a stent-avoiding and a DES-preferred strategy [32]. In the stent-avoiding group, lesion preparation was performed more frequently (71.7% vs. 51.7%, *p* < 0.05) with a rather high provisional stenting rate (48.3%). During the 12-month follow-up, the primary patency and CD-TLR rates were similar between the two groups. Thus, no clear advantage could be anticipated for a lesion preparation strategy with provisional stenting versus a preferred DES strategy in this study.

**Figure 4:**
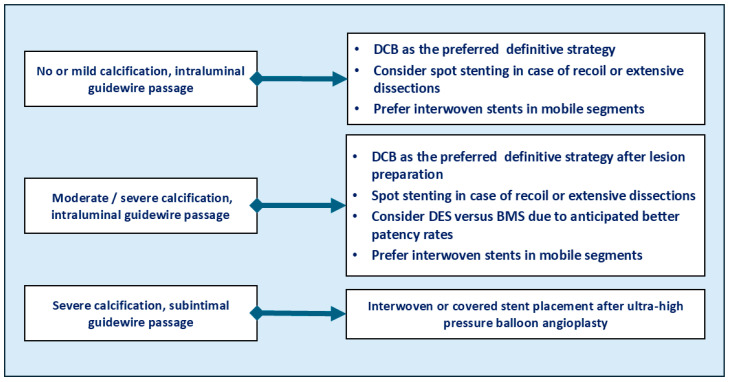
Expert algorithm for the use of the different definitive treatment strategies in femoropopliteal lesions.

Based on our previous expert consensus including a Delphi process [3], an algorithm for the use of the different definitive treatment techniques can be observed in Figure 4. Thus, calcification and the intraluminal versus subintimal guidewire passage influence not only lesion preparation but also definitive treatment strategies. Overall, DCBs are the preferred treatment strategy, complying with the ‘leave nothing behind’ principle, especially in non-severely calcified lesions or in moderately-to-severely calcified lesions past the intraluminal area and after the application of lesion preparation techniques. If the implantation of a permanent metallic implant becomes necessary due to extensive dissection or recoil, DESs or biomimetic stents may be considered and preferred to standard BMSs. Beyond these considerations based on lesion-specific parameters, treatment strategies also depend on patient-related parameters, like the RC and the patient condition and comorbidities. Thus, most operators prefer balloon angioplasty followed by stent placement to achieve prompt limb perfusion in CLTI patients, especially those with RC6 and large wounds, while lesion preparation in combination with DCB angioplasty and without stenting may be preferred in younger patients with lifestyle-limiting claudication. In addition, endovascular treatment is regarded as the first-line preferred option in patients with persistent claudication, except for complex lesions in the common femoral artery, which involve thefemoral bifurcation [33]; with CLTI, the best treatment modality needs to be selected by an interdisciplinary team, focusing not only on lesion characteristics but also on the availability of a vein graft and cardiovascular comorbidities [34]. 

## 7. Conclusions

In most cases, femoropopliteal lesions are treated endovascularly in both patients with claudication and in patients with CLTI, especially in those considered unfit for surgery. The range of definitive treatment options available for endovascular therapy is extensive, including DCBs, BMSs, DESs, interwoven stents and covered stents, and requires consideration and deep understanding of both anatomical and patient-related characteristics, including patient frailty, and cardiovascular and other comorbidities. In particular, the heterogeneity of patient and lesion characteristics highlights the lack of a one-size-fits-all solution. The implementation of a standardized treatment algorithm in daily clinical practice is therefore expected to reduce complication rates and improve patient and limb-related outcomes. In addition, most existing studies have been conducted in patients with claudication, thus leaving a knowledge gap for CLTI patients. Future studies therefore need to prospectively compare the various options in a randomized manner, including CLTI patients RC 4–6. In addition, optimal medical treatment needs to represent the cornerstone in all patients with PAD, while future studies also need to investigate the role of endovascular versus optimal conservative treatment including supervised exercise training in patients with lifestyle-limiting claudication.

## Data Availability

The data presented in this study are available on request from the corresponding author.
